# The “Empty Chairs” Approach to Learning: Simulation-Based Train the Trainer Program in Mzuzu, Malawi

**DOI:** 10.7759/cureus.1205

**Published:** 2017-05-01

**Authors:** Elaine Sigalet, Ian Wishart, Norman Lufesi, Faizal Haji, Adam Dubrowski

**Affiliations:** 1 Nursing, University of Calgary; 2 Cumming School of Medicine, University of Calgary; 3 Preventive Health Services, Ministry of Health, Malawi; 4 Peadiatrics, University of Western Ontario; 5 Emergency Medicine, Pediatrics, Memorial University of Newfoundland

**Keywords:** emergency triage assessment and treatment, malawi, simulation

## Abstract

Together, a group of Canadian colleagues from St. John's, Newfoundland, Calgary, Alberta (some via Doha) and London, Ontario introduced the first Train the Trainer in Simulation-Based Learning (TTT-SBL) program in Mzuzu Central Hospital and Mzuzu University in Malawi. The team led by Elaine Sigalet (Doha) and consisting of Ian Wishart (Calgary), Faizal Haji (London) and Adam Dubrowski (St. John's) was invited to Malawi by Norman Lufesi to conduct a two-day TTT-SBL course for facilitators who teach an Emergency Triage, Assessment and Treatment (ETAT) plus Trauma course. The following technical report describes this course.

All trainees-facilitators who took part in the first iteration of the TTT-SBL course were asked to participate in teaching an ETAT course and modify it to include elements of simulation. The new format of ETAT resulted in a reduction of time necessary to conduct the course from four days (based on historical data) to 2.5 days.

## Introduction

Despite achieving Millennium Development Goal 4, to reduce child mortality, Malawi continues to have one of the highest rates of infant and child mortality; 71 per 1000 live births in the world [1]. Since 2004, the Malawi Ministry of Health has focused on reducing the local burden of disease through the implementation of an Essential Healthcare Package (EHP).

Malawi has a population of 17. 3 million. Primary health care is delivered through health posts, community initiatives, dispensaries, maternity units, district and rural hospitals. District hospitals provide a secondary level of care and offer specialized services to patients referred from a primary health care site. Lastly, highly specialized services are only available at the central hospitals or other private specialty hospitals. Quality health services are dependent on sufficient infrastructure, service agreements with private providers, adequate numbers of trained health workers, retention of health workers and resources such as functioning equipment. Malawi health strategy 2011-2016 was developed to improve these deficiencies [2].

However, a key constraint to its successful implementation is the widespread shortage of adequately trained and available healthcare professionals in the country. This constraint impacts many developing countries and simply increasing the number of healthcare professionals has not been sufficient to address vacancies within healthcare care facilities [3]. Interestingly, a recent study by Mueller, et al. [4] indicates that upwards of 57% of all absenteeism among health workers in Malawi is attributable to providers attending in-service training and meetings away from the clinical environment [4]. Clearly, alternate strategies that facilitate continuing professional development of healthcare workers while reducing their time away from clinical duties is desperately needed.

The use of simulation-based education (SBE) is growing in developing countries [5]. Therefore, in the context of developing countries, SBE may present different needs in both the technology used and the educational strategies applied when compared to the western countries where SBE is used. In terms of technology, lower technology solutions may be more adequate and feasible. For example, computerized human patient simulators that are commonly used in the western countries are replaced with non-computerized mannequins and task trainers with verbal cueing because developing countries do not have the resources to purchase or maintain computerized equipment. Similarly, some educational strategies may need to be adapted in developing countries because of cultural nuances. For example, a form of debriefing known as advocacy and inquiry is based on the learner’s ability to reflect on their own actions which can challenge existing frameworks and standards of practice. In highly hierarchical societies, this may not be a culturally accepted form of communication between learner and the educator, and therefore may not be accepted or adhered to. Instead, direct feedback may be a culturally more acceptable form. The perceived barriers to its optimal utilization in developing countries are related to the availability of simulation technology, clinical equipment, and resources, as well as knowledge of essential educational principles that are applicable in this type of experiential learning.

This technical report describes a Train the Trainers course in SBL (TTT-SBL) that was recently conducted with Malawi health educators involved in the Emergency Triage Assessment and Treatment plus trauma program (ETAT plus Trauma) course. The implementation of ETAT has demonstrated efficacy as an educational intervention in Malawi because it has led to a substantial reduction in pediatric mortality across multiple health facilities. However, there is still room for further improvement. Local experts suspect a relationship between poor retention of skills following ETAT training and a lack of opportunity for deliberate practice during the course and as continuing education [6]. The current ETAT course is delivered over four-five days at a regional or central location, contributing to absenteeism among health workers. The TTT-SBL described in this technical report was delivered to clinical officers (n=4), nurses (n = 6) and physicians (n= 2), which reflects the population in Malawi who would be involved in the facilitation of ETAT across the country.

The ETAT + Trauma course in Malawi focuses on improving health worker’s knowledge and skill in identifying and managing children at risk for poor outcomes. In the course, this population of children are categorized as (a) those with emergency signs that need immediate intervention, (b) those with priority signs who are moved to the front of the queue and seen next, and (c) children that can be seen in order of arrival and are stable enough to wait in the queue. Children presenting with obstructed airways, compromised breathing, circulation of consciousness are deemed emergent. All children are screened for potential trauma. Children presenting at one of the dispensaries or district hospitals are usually transferred once stabilized to a central hospital for definitive care, where specialty services and equipment resides. The immediate goal of the TTT-SBL course was twofold: (a) to improve ETAT facilitators' knowledge and skills in SBE, to facilitate a shift from didactic to experiential learning within the course, and (b) to reduce the overall course length to 2.5 days, so as to limit the amount of health workers time away from their clinical duties. The long terms goal to support local sustainability was also twofold: (a) to enable a local group of ETAT facilitators to use their knowledge and skills from the course to adapt the current ETAT plus trauma course to include more use of simulation techniques, and (b) to empower this group to focus on developing a strategy to train new ETAT facilitators in simulation specific techniques.

Measurable outcomes related to the goals of the TTT SBL course will include (a) the number of revised courses run in Malawi, (b) number of participants and health care role in the courses (c) the duration of each course and (d) participant outcomes from the standardized course pre- and post-test. Comparing this data to staffing in the various clinical settings will provide information about the clinical impact of the new SBL-ETAT. Long-term measurable outcomes are also important and we suggest tracking morbidity, mortality, staff retention but realize that this will be challenging and is beyond the scope of this report.

## Technical report

The course described in this technical report was developed and conducted by four multi-professional team mentors with prior experience in facilitating similar courses in the past. The two-day course was delivered to a group of ETAT trainers in September 2016. This group of trainers was then given an opportunity to apply their newly gained skills with mentor support during facilitation of a local community ETAT course. Both courses were conducted in Mzuzu Central Hospital, Mzuzu, Malawi, which is a tertiary referral hospital for the northern region of Malawi. It was made possible by a collaboration between Mzuzu Central Hospital and Mzuzu University’s faculty of Nursing, who provided the venue, course materials, additional course facilitators, and resources to optimize course delivery.

The course was modular in nature. A blended learning approach that included didactic lectures (to build the basic knowledge, concepts and principles of teaching using simulation), expert demonstrations (to model the concepts and principles of simulation-based learning), learner led active learning (to verbalize and operationalize understanding of concepts and principles), and repeated participant led simulations (to provide opportunities for application of concepts and principles of simulation-based learning and practice) was used. For each module, as well as the course in general, learning objectives (LO) were scripted and articulated to make explicit expected learning outcomes.

During initial consultations with the local stakeholders, the team assessed the facilitators’ level of experience with simulation, as well as the availability of both simulation and clinical equipment. Facilitators had minimal experience with simulation and limited clinical equipment. To tailor the course to facilitators, limited experience with simulation, we included a module on principles of adult learning and simulation-based learning. 

To ensure the course aligned with the locally available equipment, we consulted with the Mzuzu University’s faculty of Nursing who provided the equipment. We reinforced the importance to facilitators to only use local resources. This approach was used to ensure that the facilitators were learning and practicing with the equipment available in their context. We asked facilitators to construct simulation equipment that was not available. For example, the facilitator built a cardboard vital signs monitor. The monitor was used to highlight the importance of situational awareness when the leader of a scenario needs to assume a position to monitor the team, the patient, and the monitor. However, information that would have normally be delivered via the monitor was delivered verbally. Lastly, the course faculty and at time course participants served as confederates frequently playing a mother or father.

The TTT-SBL course was developed based on the theory of mastery learning [7-8]. The course framework and each module were scripted and delivered using the following sequential approach:

1. Provide learning objectives. 2. Demonstrate the desired outcome by mentors (if applicable). 3. Break the knowledge/skills into learner appropriate steps. 4. Provide an opportunity to demonstrate application. 5. Apply knowledge/skill with mentor feedback (if applicable).

Each day of the two-day course started with an icebreaker exercise developed to introduce and increase participants’ awareness of important team performance concepts, which was an LO in each simulation scenario. Using an icebreaker to introduce team concepts was relevant for this group of learners because: (a) team performance concepts were new to learners and we needed to introduce and break the ice and (b) experiencing the concept is the best teacher; the context may change but the frustration and outcomes will be the same in real practice events when the behaviors do not reflect team performance concepts. For example, there was no role clarity in the puzzle building activity and this resulted in frustration because a few team members were bumping into each other trying to do the same task resulting in a longer time to achieve the objective. This is the same in real practice.

After the icebreaker, day one focused on enhancing trainee knowledge of education principles and effective simulation-based learning. Each module used a blended learning approach: didactic lectures to provide knowledge, expert and participant demonstrations to engage active learning. The second day focused on application and effective teamwork to apply knowledge and skills learned on day one. The concept of deliberate practice underpinned the opportunities each trainee group was given to develop and conduct a pre-brief, a simulation scenario, and facilitate a feedback session for a partner group who role played the learners. Each session was followed by the debriefing of trainees by the mentors.

### Day 1

### Module 1: Principles of adult learning

Learning Objectives:

1) Identify the characteristics of adult learners

2) Demonstrate application of principles in developing, delivering and assessing education

Content

The content of this module was focused on presenting characteristics of adult learners. Main characteristics illustrated in this module were:

- Adult learners have extensive life experiences: Learner’s needs are based on their experiences and these should be informed in the education session. Emphasis was placed on the need to use real life and clinical experiences as often as possible to teach, instead of long power point information sessions. - Realism: Realism enhances meaningfulness and relevance which is very motivating for adult learners. - Active learning: Adult learners appreciate active learning.

Our presentation approach was novel for all the participants but aimed at meeting the needs of the auditory, kinesthetic or visual learner remembering that most participants are a combination of two. Participants were asked to think about how they learned and this exercise helped them understand and appreciate this principle of using a blended learning approach. Course faculty identified the modality as participants engaged in each module. For example, when the theory was provided, faculty pointed out that participants had a visual guide, the PowerPoint presentation and auditory support from the faculty explaining the information on the PowerPoint presentation, so participants could see the deliberate approach to meet different learning styles.

### Module 2: Principles of simulation-based learning

*Learning Objectives:* 

Identify the key elements for successful use of simulation-based learning (SBL)

Content

The simulation was defined as the imitation of real practice situations or events in a specified period of time so that learners can practice application of knowledge/skills/behaviors. As applied to the developing countries, specific modalities that can be used include role-play, use of simple technology and equipment such bench top models for airways and manikins. 

Using didactic lecture format, the mentors explained that SBE has many advantages over traditional approaches to education. The simulation creates opportunities for learners to engage in deliberate practice without creating a risk to real patients [9]. As it relates to the design of teaching experiences, simulation allows for the development of a learner-centric learning environment, where the faculty member or trainer of an educational event (i.e. simulation) can control the complexity of the learning event to match learner’s current level of skills, knowledge, and attitudes. As the learner advances, the learning environment needs to become progressively more challenging [10]. Furthermore, simulation allows for the replication of rare and unusual medical events seen in real practice, thus providing opportunities for education on demand or just in time when the learner needs it [9, 11]. The simulation also creates an opportunity for trainers or facilitators to identify any performance gaps and correct them before the health care professional cares for real patients and their families [12-13].

**Figure 1:**
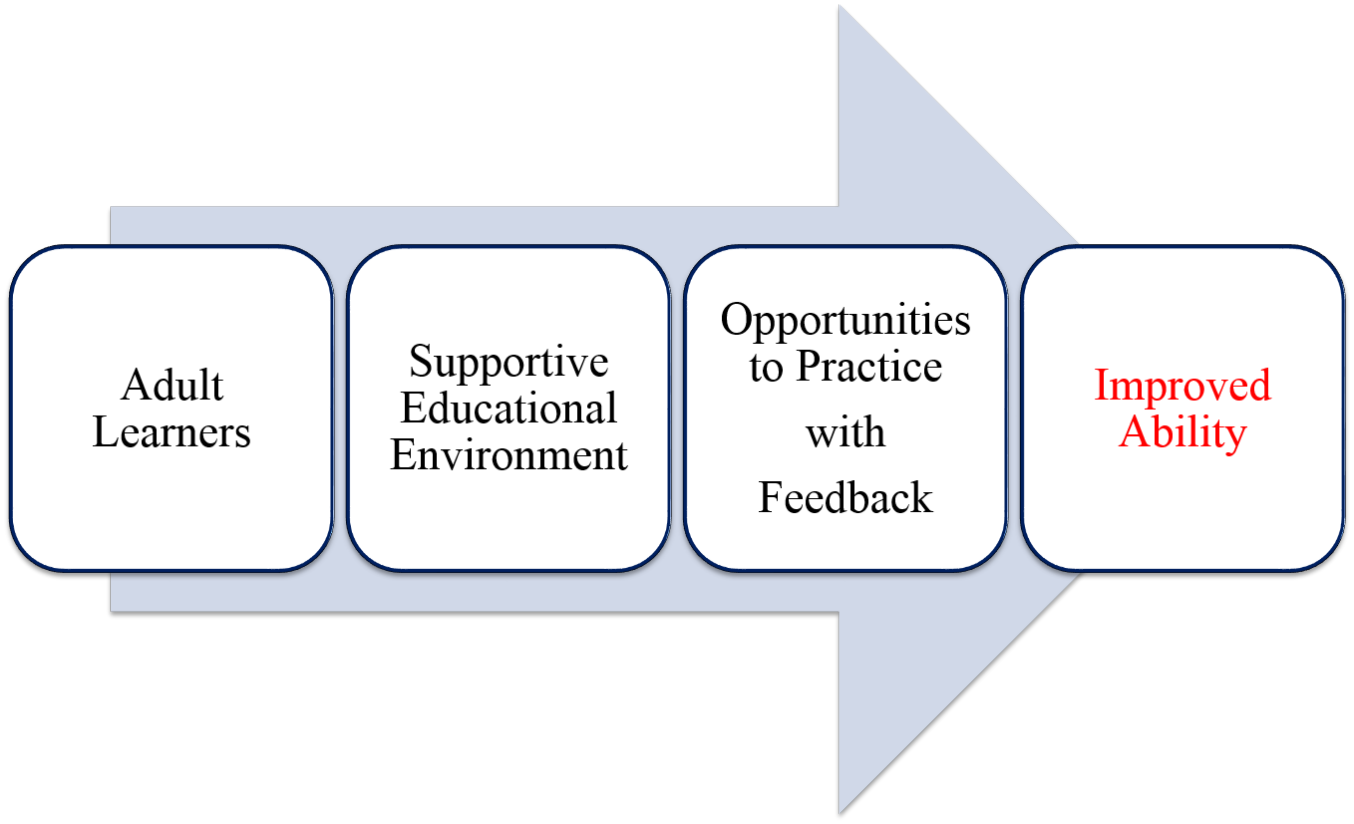
Visual representation of how simulation-based medical education improves learning

The final part of this module explored how simulation-based education works. In this section (Figure 1) was provided as a visual guide for the trainers (i.e. learners in this course).

Specifically, the lecture emphasized that simulation provides a concrete realistic experience for learners. It also provides time and guidance with reflection on action. Healthcare workers will reflect on their behaviors even when it is not facilitated but they may not have access to the right information to help reorganize thoughts which can lead to less than optimal patient management. Furthermore, simulation supports the development of new and broader understandings by helping learners reframe their thinking to align with best practice, positive experiences, and positive patient outcomes. Finally, it provides an opportunity for learners to experiment with the new knowledge, skills, and understandings.

**Figure 2:**
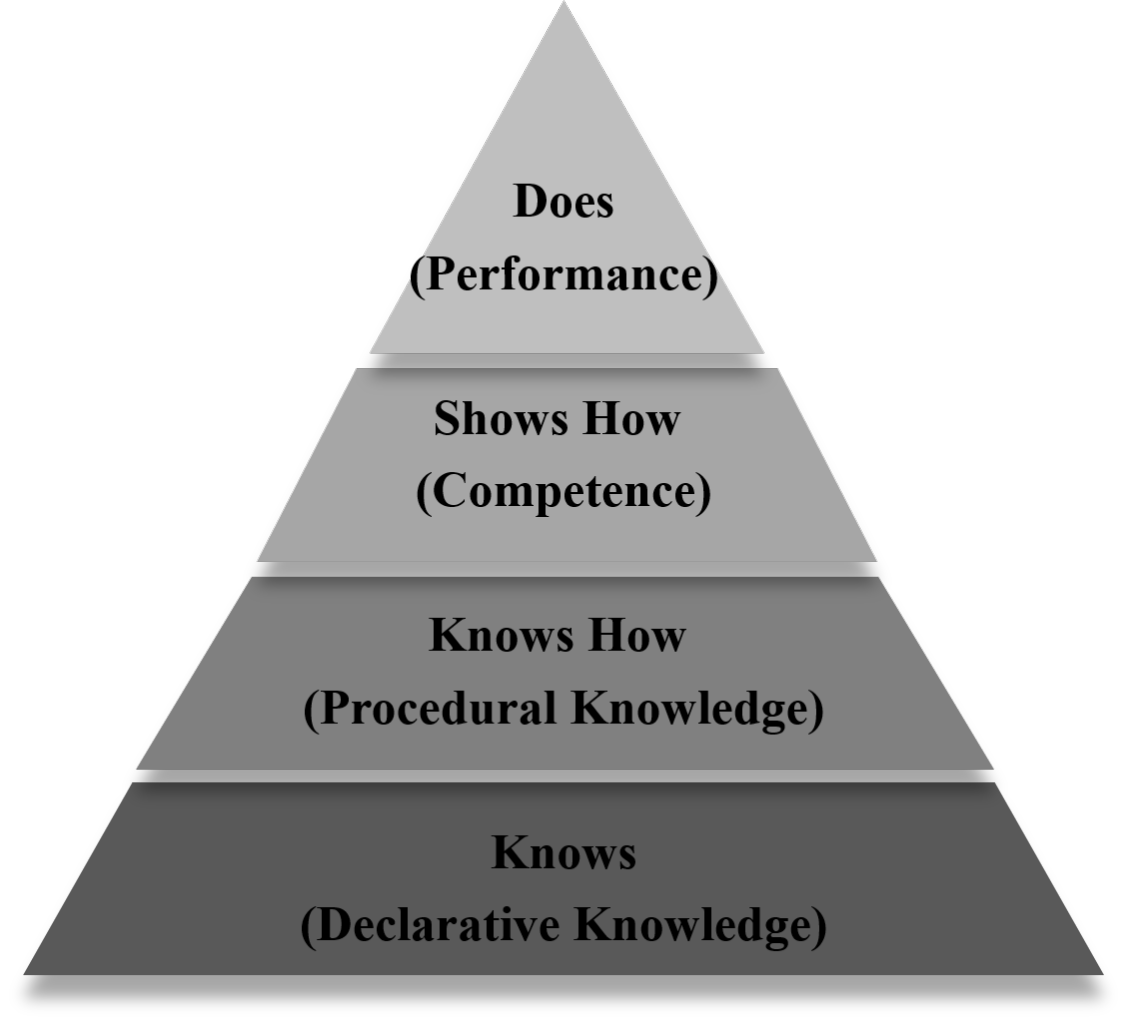
How simulation-based education fits with other forms of teaching and learning

The final part of this module explored the fit between SBE and other traditional approaches to teaching and learning. Figure 2 was provided as a visual guide for the learners.

An adaptation of an assessment metrics of clinical competence was used to illustrate where SBL fits with other forms of teaching and learning [14]. Specifically, teaching and assessing the basic knowledge happens through readings, lectures, and paper-based tests (“knows”). Simulation supports procedural knowledge and skills through the use of illustrations and demonstrations (“knows how”). Lastly, it is particularly important in the development and assessment of procedural skills and individual/team management of a patient context, which aligns with (“shows how”). In traditional approaches to education, the "shows how" occurs on real patients in the clinic environment.

### Module 3: Teaching skills

Learning Objectives: 

* *1) Identify key steps in teaching a skill

2) Develop a skill checklist

Content

This module was introduced by stating that simulation is teaching and learning tool that is used differently when teaching procedural skills, and when teaching individual or team based patient management. Module three will focus on the use of simulation for teaching procedural skills, while modules four to six will focus using simulation to teach effective patient management.

**Figure 3:**
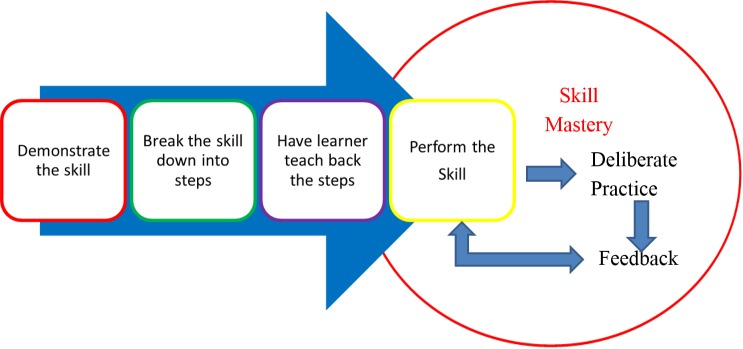
A four-step approach to skill acquisition

As illustrated in (Figure 3), the acquisition of procedural skills is optimized when a four-step approach to skill acquisition is used; demonstration, deconstruction, comprehension, and performance [15]. After an expert demonstration of the skill, it is then broken down into its basic components and individual components are demonstrated. The learner is asked to demonstrate each of the steps back to the teacher. Direct verbal feedback and if necessary physical guidance is provided at each step to support learner's success. The skill is then performed in its entirety and feedback is provided. The learner should repeatedly perform the skill with feedback until confidence is verbalized by the learner and competence is observed by the trainer. This approach will support mastery learning; transition of a novice level of ability to demonstrated competence [16].

Faculty emphasized the importance of using locally available resources for all skill stations. The following is an example of a skill taught using this approach with local resources. In Malawi, a nasal catheter is the most common mode of administration of oxygen. The distance the cannula is inserted is determined by measuring from the child’s nose to the inner margin of the eyebrow.

Skill: Insertion of a nasal cannula for oxygen administration. The skill was modeled by one of the learners. The learners worked together in their group to script each step in the procedure. At this point, the learners paired up. One person talked through the steps with their partner, then demonstrated the correct sequence of steps with the partner giving feedback. This process was repeated until the learner verbalized confidence and the partner observed competence and adherence to the sequence of steps.

Active Learning 

In this module, we have introduced elements of active learning in order for the trainers to translate the concepts learned into actions. The trainees were asked to form three-four person groups. Four groups were formed. Each group consisted of at least one nurse educator, one junior nurse, and one doctor or clinical officer. All participants were experienced in their field and this was a pre-requisite for attendance. Because this was a multi-professional course, we stratified some of the trainers from different health care profession backgrounds in order to have an inter-professional presence in each group to resonate with real practice contexts. Each group was provided with a clinical technical skill (e.g. Intravascular placement) and asked to create a skill checklist by scripting the essential steps in chronological order that are necessary to execute the skill. Trainees were told to only use equipment and resources available in the real clinical setting. Lastly, we asked trainees to use discussion and voting as consensus building mechanisms in creating a final version of a checklist for their allocated skill. 

This exercise led to the development of contextually relevant checklists that were used to both ensure standardization of steps one and two in Figure 3, as well as well as a reference for the provision of feedback during practice. The potential use of the checklist to assess procedural proficiency during learning was discussed with the group.

Next, one trainee from each group was selected to teach the procedural skill to a trainee from a different group following the developed checklist and process illustrated in Figure 3. As this was only a demonstration of principles and not a teaching and learning session, each skill was performed only twice.

### Module 4: Pre-briefing

Learning Objectives:

1) Identify the key elements of a pre-brief

2) Conduct a pre-brief with a group of learners (Day 2)

Content 

This module focused on teaching trainees how to create realistic learning environments for patient management through the use of a pre-brief scenario and guided feedback session. 

The pre-briefing phase is the first necessary step in every SBE session and it is important to prepare learners for the experience. The flow of the simulation can be often interrupted and thus its educational impact is lost if learners stop to look for, or become distracted by unfamiliar equipment and/or resources. Preparation should include the introduction of both the simulation trainers including their roles in the simulation, as well as all participants. Providing timing expectations, such as time of simulation session and feedback helps the learners maintain focus [11]. Next, the learners should be oriented to the environment and equipment and allocate some time to familiarize themselves with all equipment that will be used during the simulation. The trainers/faculty should encourage the learners to touch the manikin and handle resources so these do not become distractors in case management. Finally, ground rules need to be discussed.

Although the specific rules will vary from institution to institution and context to context, the following were suggested as possibilities and examples:

- Fiction contract

- Psychological and emotional learner safety

- Speaking out loud while working

- Duration of the simulation exercise

Faculty introduced the idea of having a basic assumption, all participants were asked to commit to thinking of their colleagues as respectful, intelligent and here to learn. This was important to learners feeling emotionally safe to take some risks in sharing during the learning activities.

Active Learning

During the active learning phase of this module, the trainees were asked to return to their groups. Within each group, the trainees were asked to come up with a single clinical case, which involved a team of health care workers and use this case to develop an SBL session. The mentors provided direct feedback on pre-briefers performance.

### Module 5: Scenario

*Learning Objectives:* 

1) Demonstrate scenario development by using the provided template to build a challenging clinical case

Content and Active Learning

For module five, the didactic content and active learning were fused. The trainees worked in their groups with their mentor to develop a relevant scenario using the provided scenario template. The template serves as an effective planning tool and pre-scenario checklist for faculty/trainers. It guides faculty or trainers to first develop Learning Objectives (LO's), prior to scenario detail. Once LO's were scripted, trainees were directed to complete the scenario stem. The stem is a short overview of the simulation scenario, similar to patient case presentation. It should contain enough information about the patient condition to prompt critical thinking in the learners so they can initiate management. The template divides each scenario into four phases to help the person running the scenario (scenario director) be prepared for effective and ineffective patient management: (a) initial presentation, (b) unstable phase, (c) worsening condition, and (d) ongoing management. Each phase is further divided into three sections to ensure the person running the scenario is familiar with (a) the clinical findings relevant to the phase of scenario, (b) the information needed to keep the learners on track because of limitations in the environment or manikin, and (c) expected to learn outcomes which should underscore scenario objectives. To orientate trainees to the template, an example was provided. We explained how to use all four phases. Trainees were told to start the scenario with an initial presentation that made the explicit need for emergent management related to the case objective. We encouraged trainees to allow teams to allocate roles before making the patient more unstable, especially when effective teamwork is an LO. The worsening phase is designed to help learners who fail to recognize emergent patient needs to get back on track. In this phase, the person running the scenario makes the patient sicker to prompt recognition and effective management. Trainees were told to think about what learners may need to see or hear to get back on track with the learning objectives. Finally, the ongoing management phase is used to show resolution of patient need when effective management is provided and prompt learners to think about next steps in definitive management.

Next, once trainees were familiar with the template and each scenario phase, they were asked to script each section of the scenario as detailed. The reality of real practice is that health workers do not always perform as trainers expect. In a simulation, this is where the scenario director can exert some control on learning. To do this effectively, the director must be prepared to change the patient presentation/clinical findings to prompt learners to deliver effective management. It was also emphasized that in real practice, phase one and phase two could be fused depending on the level of the learner and the LO's. Lastly, we told trainees that the learners could progress quickly through phase one, two and four when learning objectives are easily achieved and competent patient management is observed.

Once the scenario phases were scripted, learners were asked to identify the resources they need to create realism. This section of the template provides choices that were scripted to match the types of resources that are available and used in Malawi. 

The final section requires trainees to identify the number of faculties that would be needed to effectively run the scenario. Here we acknowledged that in current conditions, limited-resource environments, the scenario director (the trainer) would need to set up the resources, direct the scenario and provide the feedback. In courses run by more than one trainer, there may be more faculty to help especially when groups are paired. Although participant roles did not have a dedicated section on the template, trainees were encouraged to keep learners in their real role. Nurses should play nursing roles and clinical officers and physicians play the leader of the event. The exception to this occurs when there is not a learner for the role or the LO focuses on familiarization of other team roles. Trainees were encouraged to keep learners in their real roles. Furthermore, faculty emphasized the importance of mimicking the real practice setting with roles. For example, in the emergency room in the district hospital, there is usually just one triage nurse and the clinical officer has to be called. Other nursing support would be drawn from an in-patient unit.

### Module 6: Feedback

Learning Objectives: 

1) Identify three methods for effective feedback

2) Demonstrate the ability to use at least two of the three methods

Content

Next, to practice, feedback is the most important step in the learning process [16]. During this phase of learning, the trainer or facilitator who provides the feedback attempts to close performance gaps that were observed during practice. There are three ways to provide feedback [17].

The first way is to be direct. The trainers are encouraged to tell the learner what they did wrong and how to do it right. When the plus/delta and advocacy and inquiry techniques are used, the feedback phase is typically referred to as the debriefing phase because these techniques engage discussions with the learners or two-way exchange of information giving the learner an opportunity to share the thoughts that led to their actions. Specifically, for the plus/delta approach, the plus stands for what went well and the delta refers to what needs to change or be done differently next time because it did not lead to the expected outcome. The second technique is more difficult for novice trainers or facilitators, the advocacy and inquiry technique involves checking assumptions. It relies heavily on observations or restating what was said or done, an honest appraisal of concern or appreciation and provides an opportunity for the learner to share their perceptions or thoughts that led to the actions so they can be discussed further. Because the advocacy and inquiry technique can be challenging to novice trainers, we have developed a set of prompting sentences and questions that they may use:

“I observed that (in-non-judgemental way, state the performance that highlighted the performance gap).” I was concerned that (in the non-judgemental way, state what could be some of the implications), I was wondering how you saw it?”

NOTE: This technical report is not intended to be an in-depth review of plus/delta or advocacy and inquiry techniques to debriefing. For in-depth descriptions, the readers are encouraged to read: Cheng, et al., 2011 [18]; Rudolph, Simon, Rivard, Dufresne, & Raemer, 2007 [19].

**Figure 4:**
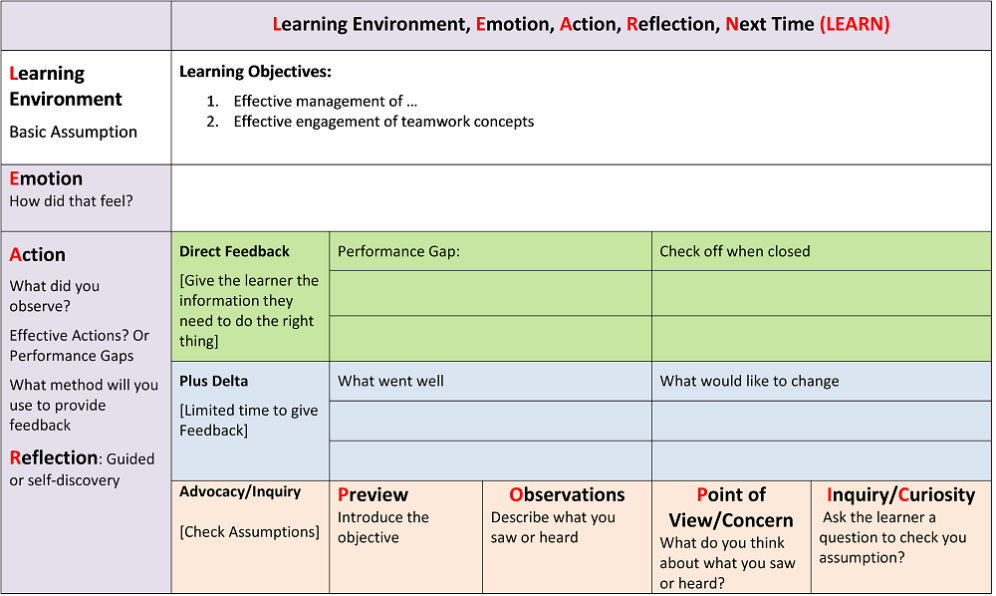
LEARN framework

Once the trainees were familiarized with the concepts underpinning effective feedback, the feedback process was described. We used the learning environment, emotion, action, reflection, next time (LEARN) framework (Figure 4) in order to help the trainers formulate a feedback plan [20]. The LEARN framework was developed to help trainers organize an effective SBE feedback session. To support effective use of the cognitive tool, the learners are asked to leave the simulation environment for few minutes or if this is not possible, ask the learners to refrain from discussing the scenario until prompted by the trainer. The rationale behind this strategy is to allow trainers an opportunity to formulate their feedback plan following the LEARN steps.

As illustrated in Figure 4, in step L (learning objectives), the trainers need to revisit the learning objectives in light of the observed performance gaps. In step E (emotions) the trainers should ask the learners to express any emotions associated with the simulation. Giving learners an opportunity to express emotion can be helpful. For many learners, it will allow them to now focus on discussion and learning in the next step. This is easy to accomplish at the start of any feedback session by asking the learners to share how they thought the scenario went or how they felt managing the scenario. This also gives the trainers important insight into the learners’ needs as important concepts that created frustration and are often expressed and can be used to steer next step of feedback. In step A and R (actions and reflection), one of the previous feedback techniques can be used:

- Direct feedback to close the gap in knowledge/skill/behavior

- Plus/delta to explore the concept further as a group

- Advocacy and inquiry to try and explore thoughts/thinking that led to actions, so feedback can be more focused on closing the performance gap and probably more meaningful to the learner

These different approaches can be used interchangeably or in tandem within the same feedback session. For example, if advocacy and inquiry approach leads to a discussion which does not facilitate closing the performance gap, the trainer is encouraged to use a direct feedback approach to ensure that the gap is closed. This is very important for novice trainers as they can perseverate on getting the active learning (A/I) right and can forget to close the performance gap.

The session ends with step N (next steps), during which the trainer is instructed to ask the learners about providing one thing that they have learned from the session. Learners’ responses should follow the performance gaps noted in steps E and A and should align with the learning objectives articulated in step L. This last phase should also prompt the trainer to give the learners a second opportunity to manage the scenario and to experience the value of application of new understanding and knowledge to support freezing better practice concepts. Learners who leave feeling effective in their role will be motivated to engage in other SBE [21].

Active Learning

The trainees were asked to return to their groups and a mentor was assigned to each group. They were provided with four scenario vignettes (Table 1). Each vignette had a few strategic embedded performance gaps. As a group, the trainees were asked to identify the performance gaps in each vignette. Next, each participant was given five minutes to formulate the feedback plan following the LEARN framework and using at least two of the three feedback techniques to provide feedback to the mentor who would role play the health worker with the performance gap. The mentor provided them with direct feedback on their performance. The other trainees were allowed to contribute in the feedback session after the initial feedback was given to the trainee from the mentor.

**Table 1:** Patient vignettes

Vignettes
Sharita is nine months old and weighs seven kg. Her mother brings her to the clinic because she has had diarrhea for a week. The mother tells the health worker that Sharita is no longer breastfed, and is too tired to drink from a cup. The health worker assesses Sharita. He finds that she is breathing fast and has some work of breathing. He continues to look at circulation and notes that her hands are cold and her pulse is fast and weak. He thinks she might be in shock. He finds her to be lethargic with sunken eyes, and a skin pinch goes back very slowly. He also thinks she is very dehydrated but not malnourished.
A four-year-old male hit by a bicycle was carried in on a blanket. The child was unconscious responding only to pain. His breathing was noisy. The health worker calls immediately for the senior health worker to help as he takes the child from the father and carries him into the emergency treatment room. The senior health worker immediately puts the child on oxygen to support his noisy breathing, starts an IV and starts to give a bolus of normal saline for fluid. The junior health worker gets a warm blanket to cover the child. Once the fluids are infusing the senior health worker asks the junior health worker about the child’s history.
In triage of a 10-year-old male who was rushed to emergency after falling from a coconut palm half an hour earlier, you find his hands are cold and the capillary refill time is longer than three seconds. The first health worker recognizes the child needs immediate intervention because of the cold hands and brings the child directly into the emergency treatment room and calls for help from the one seniors who is currently seeing another child on the pediatric unit. The senior does not come down for 15 minutes as he was called to see another child who needed ventilation support on the unit and is just stabilizing that patient. When the senior arrives he is verbally upset with the junior because the only notable intervention was the initiation of oxygen by nasal cannula at two liters and two obvious unsuccessful IV attempts. The father is watching the exchange between the workers.
Dano is eight months old and weighs six kg. He has had diarrhea for a week and is very sick. The clinical officer recognizes that the child is breathing adequately and he does not think he is in shock. The health worker sees that Dano's eyes are sunken. When encouraged, Dano is able to take a sip of water, but drinks poorly. A skin pinch goes back very slowly. The health worker finds Dano has diarrhea with severe dehydration. He is also severely malnourished. The health worker worried about the dehydration puts the child on one-two liters of oxygen by nasal cannula and starts an IV. The health worker gives the child 60 cc of normal saline very quickly.

### Day 2

### Active learning

The second day of the TTT-SBL course was dedicated to the delivery of the trainer generated simulation scenarios. One group was tasked with directing the scenario and feedback session whilst the other group role played the learners. The trainers delivering the scenario were asked to follow the pre-brief, scenario, feedback format. Trainers were given an hour to gather all necessary equipment, set up the environment, and practice the scenarios (i.e., dry-run). Specifically, each scenario was designed to focus on common challenges for ETAT staff. These challenges were identified in consolation with local leadership. Vignette A depicted a full airway, breathing, circulation, disability, dehydration, exposure (ABCD) triage for a child presenting with emergency signs at circulation. Vignette B depicts two performance gaps: failure to initiate trauma management and failing to identify and relate trauma history to the clinical officer. Vignette C depicts three performance gaps: failure to initiate trauma management, failure to identify urgency of the situation to the clinical officer or activate plan B, and aggressive communication in front of the father. Vignette D depicts three performance gaps: incomplete triage identifying findings for A and C, failure to initiate management for a severely malnourished child which would use oral rehydration salts instead of intravenous fluid, and if Intravenous fluids no bolus.

Upon completion of the first simulation scenario, group roles were reversed and a second scenario was conducted. Each simulation consisted of a five-minute pre-brief, 15-minute scenario, and 30-minute feedback phases. The trainers were encouraged to use all of the three feedback techniques presented on day one. At the end of each scenario, the two mentors of the paired groups provided direct feedback to the trainees delivering the SBE. For feedback, the mentors used the visual guides (Figures 4) to illustrate the performance gaps and expert model course theory and skills.

After lunch, the same format was repeated following exactly the same schedule and using exactly the same scenarios. This gave each group a chance to apply their learning. The day and the course concluded with a course evaluation session.

## Discussion

The purpose of this technical report was to describe a two-day TTT-SBL. The immediate goals of the course were (1) to improve ETAT facilitators’ knowledge and skills in SBE to facilitate a shift from didactic to experiential learning, (2) whilst reducing the course duration from the current four to five days to 2.5 days. The long terms goal was to enable to ETAT facilitators who received the training to form a working group to review and insert more SBL into the current ETAT course. This group will focus on developing a strategy to train new ETAT facilitators in simulation specific techniques.

This technical report was not intended to provide an in-depth review of all the concepts presented. Instead, it was written to provide a conceptual framework and visual aids to assist the development of a similar course in other developing countries and regions. Therefore, it is pertinent to the success of future iterations and adaptations of this course that the mentors (i.e. faculty developing and conducting this course) have in-depth expertise within each of the areas described.

## Conclusions

The long-term aim of this initiative is to decrease morbidity and mortality through the introduction of a context specific faculty development program focused on pedagogy and administration of SBL programs. We acknowledge that linking the use of SBL in ETAT to child morbidity and mortality is beyond the scope of this technical report, but should be considered in future research efforts related to both SBE, ETAT and child outcomes. We anticipate that an ETAT course underpinned by SBE will enhance the capacity and quality of health service delivery in developing countries. Health workers taking an SBL ETAT should experience fewer performance gaps in practice settings. Increasing hands-on opportunities in training in the western world with SBE has led to better individual competence and team performance positively impacting patient outcomes [6, 12]. It will be important to examine this potential in developing countries. In addition to potential benefits on infant and child mortality, optimization of current ETAT courses and trainer abilities holds potential for decreasing the course duration to 2.5 days. The course was originally developed to be taught in 3.5 days and was often taught in four-five days in Malawi and other developing countries. With the current challenge of training health workers and inadequate staffing too, there is a critical need for shortening courses like ETAT to enhance both quality and capacity of health service delivery in these countries.
